# Pulsed direct and constant direct currents in the pilocarpine iontophoresis sweat chloride test

**DOI:** 10.1186/1471-2466-14-198

**Published:** 2014-12-13

**Authors:** Carla Cristina Souza Gomez, Maria de Fatima Servidoni, Fernando Augusto de Lima Marson, Paulo Jose Coelho Canavezi, Adriana Mendes Vinagre, Eduardo Tavares Costa, Antonio Fernando Ribeiro, Maria Angela Gonçalves de Oliveira Ribeiro, Adyleia Aparecida Dalbo Contrera Toro, Celia Regina Pavan, Michelle Vivine Sá dos Santos Rondon, Sonia Leticia Silva Lorena, Francisco Ubaldi Vieria, Jose Dirceu Ribeiro

**Affiliations:** Departament of Pediatrics, Faculty of Medical Sciences, State University of Campinas, 13083-887 Campinas, SP Brazil; Centre for Research in Pediatrics (CIPED), Faculty of Medical Sciences, State University of Campinas, 13083-887 Campinas, SP Brazil; Gastrocentro - Endoscopy Unit - State University of Campinas (Unicamp) - Cidade Universitária Zeferino Vaz - Barão Geraldo, 13083-872 Campinas, SP Brazil; Departament of Medical Genetics, Faculty of Medical Sciences, State University of Campinas, 13083-887 Campinas, SP Brazil; Center for Biomedical Engineering (CEB), State University of Campinas, 13083-970 Campinas, SP Brazil; Department of Biomedical Engineering, Faculty of Electrical and Computer Engineering, University of Campinas, 13083-881 Campinas, SP Brazil; Federal Institute of Education, Science and Technology of Sao Paulo (IFSP), Campus Campinas, 13069-901, km 143,5, Campinas, SP Brazil

**Keywords:** Constant direct current, Cystic fibrosis, Iontophoresis, Pulsed direct current, Sweat test

## Abstract

**Background:**

The classic sweat test (CST) is the golden standard for cystic fibrosis (CF) diagnosis. Then, our aim was compare the production and volume of sweat, and side effects caused by pulsed direct current (PDC) and constant direct current (CDC). To determine the optimal stimulation time (ST) for the sweat collection. To verify the PDC as CF diagnosis option.

**Methods:**

Prospective study with cross-sectional experimental intervention. Experiment 1 (right arm): PDC and CDC. ST at 10 min and sweat collected at 30 min. Currents of 0.5; 0.75; 1.0 and 1.5 mA and frequencies of 0, 200, 1,000 and 5,000 Hz applied. Experiment 2 (left arm): current of 1.0 mA, ST at 5 and 10 min and sweat collected at 15 and 30 min with frequencies of 0; 200; 1,000 and 5,000 Hz applied Experiments 1 and 2 were performed with current density (CD) from 0.07 to 0.21 mA/cm^2^. Experiment 3: PDC was used in typical CF patients with two *CFTR* mutations screened and or with CF diagnosis by rectal biopsy and patients with atypical CF.

**Results:**

48 subjects (79.16% female) with average of 29.54 ± 8.87 years old were enrolled. There was no statistical difference between the interaction of frequency and current in the sweat weight (p = 0.7488). Individually, positive association was achieved between weight sweat and stimulation frequency (p = 0.0088); and current (p = 0.0025). The sweat production was higher for 10 min of stimulation (p = 0.0023). The sweat collection was better for 30 min (p = 0.0019). The skin impedance was not influenced by ST and sweat collection (p > 0.05). The current frequency was inversely associated with the skin impedance (p < 0.0001). The skin temperature measured before stimulation was higher than after (p < 0.0001). In Experiment 3 (29 subjects) the PDC showed better kappa index compared to CDC (0.9218 versus 0.5205, respectively).

**Conclusions:**

The performance of the CST with CDC and PDC with CD of 0.14 to 0.21 mA/cm^2^ showed efficacy in steps of stimulation and collection of sweat, without side effects. The optimal stimulation time and sweat collection were, respectively, 10 and 30 min.

**Electronic supplementary material:**

The online version of this article (doi:10.1186/1471-2466-14-198) contains supplementary material, which is available to authorized users.

## Background

The first report of increased levels of sodium and chloride on sweat in cystic fibrosis (CF, MIM #219700) patients was published in 1953 [1]. Later, Gibson and Cooke [2] described a method of cholinergic stimulation with pilocarpine iontophoresis on the skin to facilitate the dosage and stimulation of the sweat electrolytes. This method is known as the classic sweat test (CST), and still is considered the golden standard for CF diagnosis [3, 4].

In the sweat gland ducts, chloride ions (Cl-) are reabsorbed by the CFTR (cystic fibrosis transmembrane regulator) [5]. Then, in health subjects, the normal concentration of sweat chloride levels are minor than 30 mmol/L [6, 7]. CF patients show in the distal ducts of sweat gland, absence or defect of CFTR protein, and reduction of Cl- reabsorption [8, 9].

Two distinct CST confirms CF diagnosis when sweat Cl- levels are greater than 60 mmol/L. The sweat test by European perspective is divided by ranges considering the following groups: minor than 30 mmol/L (normal range), 30 to 60 mmol/L (intermediate range), higher than 60 mmol/L [6, 7, 10].

The CST implementation presents many difficulties. It is divided into three stages: stimulation, collection and electrolytes measurement [11]. During the stimulation fase can occur: low amount of sweat production, difficulties to immobilize children, stress, burns, pain, hives, redness and skin irritation, when the current density (CD) is applied over than 0.5 mA/cm^2^. Electric shock and skin injuries may occur when the metal electrode is in direct contact with the skin, in case of misuse. In collection stage can occur: contamination, sample evaporation and inadequate time in the sweat production. For electrolytes dosage the problems include a lack of specialized professional to perform the sweat analysis and variability of equipment brands for electrolytes measurement [2, 11, 12].

Taking into account the difficulties in CST realization, to standardize the CST in Brazil has been a great problem. Sweat stimulation equipments are, principally, constructed by CF reference centers and have no authorization from the national health surveillance agency. Recently, the Brazilian government authorized the Macroduct® (Wescor®, USA, Utah) system for the CST [13–15]. However, due to the difficulties in purchasing and maintaining the devices, few Brazilian laboratories have this equipment, with no standard use.

In the literature, the constant direct current (CDC) is used to induce the sweat by iontophoresis. There are reports on the efficiency in the substances delivery through the skin by pulsed direct current (PDC), without causing burning and discomfort risk when compared to CDC. On the other hand, some authors have found greater, smaller or equal efficacy between CDC and PDC for substances delivery into the skin by iontophoresis [16–19].

Sweat test made with gauze or filter paper use the minimum amount of 75 mg of sweat (classical experiment by Gibson and Cooke), while the sweat collected by serpentine or plastic capillary tube is 15 μL. After collecting, sweat samples are sent to dosage the electrolytes concentration [2, 6, 7, 13].

The aim of the present study was to compare the sweat weight and side effects by PDC and CDC and determine the optimal stimulation time of sweat collection in healthy individuals. In addition, we checked PDC use for CF diagnosis in patients with and without confirmed CF diagnosis, who had previously performed CST with CDC.

## Methods

Experimental study, with prospective cross-sectional intervention in subjects with and without CF disease.

For the CST, the sweat-inducing device developed by the Centre of Biomedical Engineering University was used. The iontophoresis device is portable, easy to use and able to select PDC and CDC, with triangular waveform, frequency from zero to 5,000 Hz, maximum current of 5 mA, software settings and data acquisition support, and two electrodes comprising 70% copper and 30% zinc with diameter of 30 mm. The battery powered was used in the sweat test device. Sweat tests were performed with 0.5% pilocarpine (refrigerated when not in use).

The Research Ethics Committee of the State University of Campinas (#80430/2012) approved the project. All subjects read and signed an informed consent.

The room temperature was stabilized at 25°C and the applied CD was calculated according to the formula P = I/S; P = density, I = current and S = electrode area.

The variables gender, ethnicity, age and body mass index (BMI) were considered. The BMI calculation was performed by the formula: weight (kg)/height^2^ (m) [20].

Three sets of experiments were performed:

### Experiment 1: right arm of healthy subjects (randomly selected frequency and intensity of the current)

Objective: to compare the sweat weight and impedance of the electrode assembly, gauze and skin between CDC and PDC. We used stimulation time of 10 min and sweat collection of 30 min. We applied currents of 0.5; 0.75; 1.0 and 1.5 mA, frequency of 0, 200, 1,000 and 5,000 Hz. Data collection was done along six months.

### Experiment 2: left arm of healthy subjects (randomly selected stimulation time, collection time and current frequency)

Objective: to compare the sweat weight and impedance of the electrode assembly, gauze and skin with different stimulation and collection times. Were applied fixed current of 1 mA in the tests. Stimulation time of five and 10 min, and collection at 15 and 30 min at frequencies of 0, 200, 1,000 and 5,000 Hz. Data collection was done along six months.

### Experiment 3: right arm of patients with classical and atypical CF (PDC current of 1 mA, frequency of 1,000 Hz, stimulation time of 10 min and collection time of 30 min)

Objective: to compare the sweat weight and chloride levels by PDC and CDC. Compare the CF diagnosis by CST (considering the PDC and CDC) with the results of rectal biopsy [21] and *CFTR* mutations. Data collection was done during two days for all subjects enrolled. All the subjects enrolled are attended in CF reference center considering the clinical symptoms and comorbidities related with CF disease. The subjects performed the CST – CDC before the study, and PDC during the study. The data was compare with *CFTR* mutations and CFTR expression and function (determined by rectal biopsies).

The CST was performed in three steps: stimulation, collection and measurement, according to Gibson and Cooke [2], with a distance of five cm between the electrodes.

We evaluated the discomfort in the first, fifth and last minute by the visual analog scale (VAS) (Additional file 1) [22].

The impedance of the set composed by electrode, gauze and skin during the stimulation time was calculated by Ohm’s law [Z = V_RMS_ / I_RMS_ (Ω)]; Z = impedance (Ω); V_RMS_ = voltage measured by RMS (V); I_RMS_ = current measured by RMS (A).

To collect the sweat were used two filter paper of 17.5 cm^2^ covered with plastic and bandage crepe filter. In classical Gibson and Cooke test, the 75 mg sweat criteria was considered, being 1 g (sweat)/m^2^ (sweat collection area)/min (collection time) in a area of 5.5 cm diameter filter paper. To simulate this value, a sweat weight of 54 mg was considered in our data.

Manual titration and flame fotometometria were used to analyze chloride and sodium concentration in mEq/L, respectively [2, 6, 7]. Quality controls (positive cases of sweat tests and health subjects) were used to perform the chloride and sodium dosage.

Each subject performed Experiment 1 and Experiment 2 at the same time. The Experiment 3 was performed after the conclusion of the Experiments 1 and 2.

### Statistical analysis

SAS (Statistical Analysis System) for Windows, vs 9.2. (SAS Institute Inc, 2002–2008, Cary, NC, USA) was used for data analysis. Graphs were designed using SPSS (Statistical Package for the Social Sciences) vs 22.0 (IBM®, Armonk, NY, USA) and Medcalc vs 13.2.2 (MedCalc Software, Acacialaan 22, B-8400 Ostend, Belgium).

Numerical variables are shown by position and dispersion measures. Analysis of variance (ANOVA) for repeated measures was used to compare data parameters. We applied the transformation of posts due to the lack of normal distribution.

Spearman’s correlation was applied to correlate the chloride values and sweat weight for CDC and PDC of CF patients, atypical CF patients, and healthy subjects. Kappa index was performed to measure the concordance among data.

The significance level was set at α = 0.05 and β = 0.08. Results were presented in tables and graphs.

## Results

The study enrolled 77 subjects, 48 healthy individuals (79.16% female) with an average of 29.54 ± 8.87 years and 29 patients, with classical and atypical CF, with an average age of 24.37 ± 12.11 years. Table 1 shows the features from the studied population including the experiments 1, 2 and 3. *CFTR* mutation frequency and mutation characterization are described, respectively, in the Additional files 2 and 3.

**Table 1:** **Clinical characterization of subjects enrolled in the experiments 1, 2 and 3**

Variable	Sample size	Distribution
**Experiment 1 and 2 subjects**		
Sex (masculine)	48	18/48 (37.5%)
Etnicity (Caucasian)	48	35/48 (72.9%)
Age*	48	29.54 ± 6.51; 27 (20 – 55)
Height*	48	167.92 ± 8.74; 166 (150 – 191)
Weight*	48	67.21 ± 9.77; 65 (50 – 95)
Body mass index*	48	23.83 ± 2.56; 23.22 (18.14 – 34.48)
**Experiment 3 subjects**		
Sex (masculine)	29	10/29 (34.5%)
Etnicity (Caucasian)	29	25/29 (86.2%)
Age*	29	24.37 ± 12.106; 21 (6 – 46)
Height*	29	1.56 ± 0.154; 1.58 (1.22 – 1.87)
Weight*	29	50.078 ± 13.81; 52 (26.8 – 76.6)
Body mass index*	29	20.21 ± 3.60; 19.71 (13.20 – 29.65)

*Mean ± standard deviation; median (minor and major values).

### Experiment 1 – testing intensity and frequency of the current for sweat test

**Figure 1:**
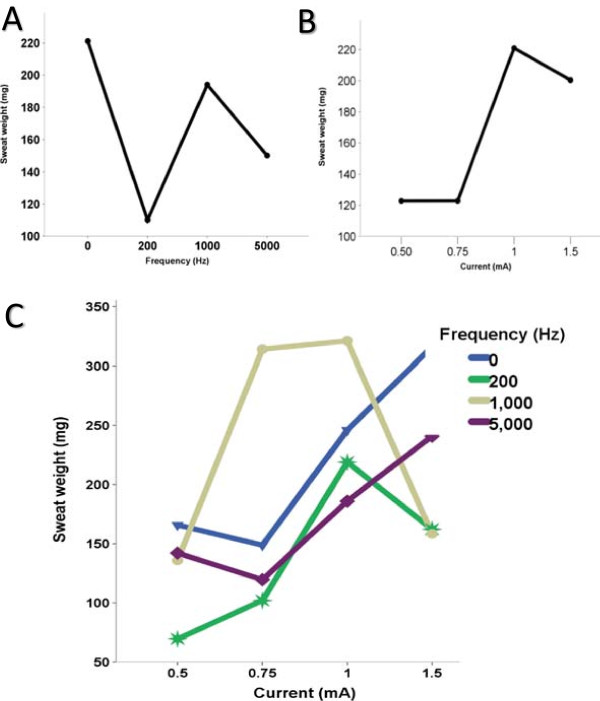
**Association with the sweat weight, current and frequency. A**. Current values and sweat weight (p = 0.0088). **B**. Frequency values and sweat weight (p = 0.0025). **C**. Interaction among current, frequency and sweat weight (p = 0.7488).

There was no statistical difference between the interaction of frequency and current for the sweat weight (p = 0.7488) (Table 2, Figure 1).

**Table 2:** **Sweat weight (mg) at different frequencies and current**

Frequency (Hz) ^*^	Current (mA) ^#^			
	0.5	0.75	1	1.5
0 (CDC)	167 ± 5.9	173 ± 12.2	236 ± 3.6	328 ± 11.8
200	78 ± 2.0	91 ± 3.2	189 ± 5.5	150 ± 4.2
1000	146 ± 8	254 ± 11.1	300 ± 13.7	226 ± 16.6
5000	136 ± 3.2	125 ± 1.2	221 ± 9.0	212 ± 5.7

CDC, constant direct current; Hz, Hertz, mA, milliampere. *For the frequency analysis and sweat weight, p-value = 0.0088; ^#^For the current and sweat weight, p-value = 0.0025; for interaction between current, frequency and sweat weight, p-value = 0.7488.

Considering the CST by Gibson and Cooke, 16.7% (3/24) of the subjects in the Experiment 1 had no sufficient sweat weight (<75 mg) for low frequency versus 4.2% (1/24) for high frequencies, but no significant association was achieved (p = 0.348). However, considering the 54 mg sweat criteria in our data, all the subjects sweated correctly to perform the sweat test.

There was association from sweat weight with current frequency (p = 0.0088, Figure 1b) and with current intensity applied (p = 0.0025, Figure 1a).

### Experiment 2 – testing stimulation time, collection time and the frequency of the current

High sweat production occurred with 10 min of stimulation compared to 5 min (p = 0.0023), and 41.7% (10/24) of subjects had insufficient sweat weight (< 54 mg) in 5 min versus 4.2% (1/24) for 10 min (p = 0.002).

**Figure 2:**
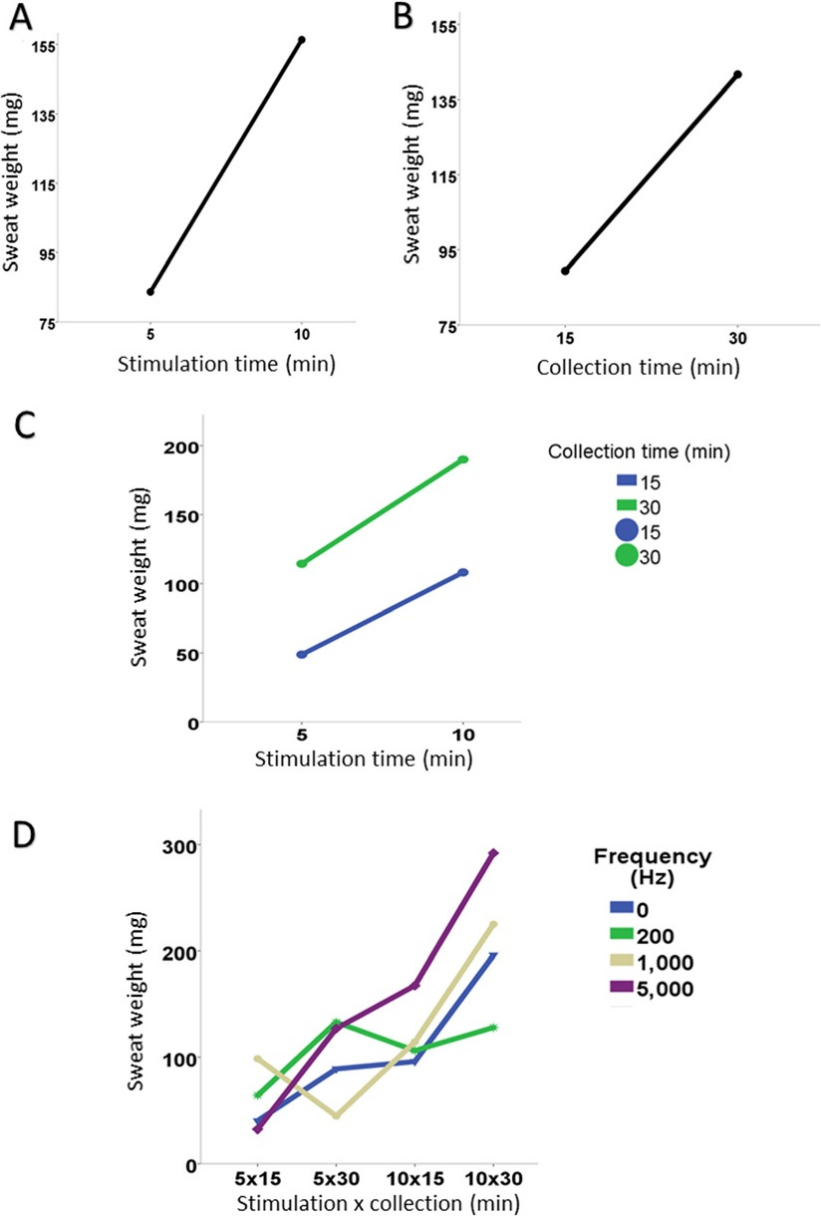
**Association of sweat weight with the stimulation and collection time and frequency. A**. Stimulation time and sweat weight (p = 0.5898). **B**. Collection time and sweat weight (p = 0.9320). **C**. Stimulation and collection time and sweat weight (p = 0.1204). **D**. Stimulation and collection time, frequency and sweat weight (p = 0.7578).

The collection time of 30 min allowed greater sweat weight than 15 min (p = 0.0019) (Table 3, Figure 2), and 37.58% (9/24) of subjects had insufficient sweat weight (< 54 mg) in 15 min versus 8.3% (2/24) for 30 min (p = 0.018).

**Table 3:** **Sweat weight (mg) for frequencies and stimulation and collection time of sweat, with fixed current of 1 mA**

Frequency (Hz) ^*^	Stimulation Time x time of collection of the sweat ^#^			
	10 × 30	10 × 15	5 × 30	5 × 15
0 (CDC)	160 ± 5.1	76 ± 4.8	18 ± 0.204	41 ± 0.9
200	137 ± 3.1	118 ± 14.4	147 ± 0.04	77 ± 3.9
1000	222 ± 7.4	138 ± 7.0	83 ± 0.09	80 ± 4.9
5000	267 ± 7.3	164 ± 8.8	111 ± 0.03	69 ± 6.3

CDC, constant direct current; Hz, Hertz, mA, milliampere. *In the analysis considering stimulation time, p-value = 0.0023 (10 > 5 min); ^#^In the analysis considering the collection time, p-value = 0.0019 (30 > 15 min). For frequency (p = 0.4672), frequency + stimulation time (p = 0.3046), frequency + collection time (p = 0.6723), time of stimulus + collection time (p = 0.7131) and frequency + collection time + stimulus time (p = 0.7322), there was no statistical association.

The skin impedance was not influenced by the time of sweat stimulation (p = 0.2433), and sweat collection (p = 0.2697). There was no influence of interaction between stimulation and collection time (p = 0.1204).

The current frequency was associated inversely with skin impedance (p < 0.0001). Moreover, the current frequency showed no association with the collection time (p = 0.7578), as well with the stimulation time (p = 0.5898). There was no association between stimulation and collection time (p = 0.9320) (Tables 4 and 5).

**Table 4:** **Impedance data (in Ohm) for frequencies and currents, with a fixed time of 10 min for stimulation and 30 min for collection**

Frequency (Hz) ^*^	Current (mA) ^#^			
	0.5	0.75	1	1.5
0 (CDC)	11303.4 ± 5487.4	11339.3 ± 7507.5	8178.2 ± 2004.9	5685.5 ± 838.5
200	8615.9 ± 4763.1	6086.4 ± 1278.4	5995.3 ± 1592.9	4727.6 ± 562.7
1000	6787.3 ± 1997.8	4387.7 ± 703.0	4198.1 ± 2125.4	2975.2 ± 216.2
5000	6221.3 ± 1962.3	4480.2 ± 450.5	4462.3 ± 1848.2	3226.1 ± 159.6

CDC, constant direct current; Hz, Hertz, mA, milliampere. *In the analysis considering the frequency in relation to the impedance, p-value = 0.0007; ^#^In the analysis considering the current in relation to the impedance, p-value = 0.0003; for interaction between current and frequency in relation to the impedance, p-value = 0.9871.

**Table 5:** **Impedance (in Ohm) with frequency data for each diade of stimulation and collection time, with fixed current of 1 mA**

Frequency (Hz)	Stimulation time* × collection time on sweat test ^#^			
	10 × 30	10 × 15	5 × 30	5 × 15
0 (CDC)	7656.7 ± 363.1	7527.3 ± 3199.3	9856.1 ± 2517.7	13030.4 ± 2641.0
200	6765.5 ± 1271.5	5500.3 ± 697.5	5681.9 ± 1075.2	5795.6 ± 1457.4
1000	4822.4 ± 1280.9	3895.6 ± 117.2	4965.8 ± 613.7	4356.3 ± 882.2
5000	5317.9 ± 831.4	5330.1 ± 3088.6	5376.0 ± 1691.7	5873.9 ± 74.6

CDC, constant direct current; Hz, Hertz, mA, milliampere. *In the analysis considering time of stimulation, p-value = 0.2433; ^#^In the analysis considering the time of collection, p-value = 0.2697. There was no statistical association for frequency (p <0.0001), frequency + stimulation time (p = 0.5898), frequency + collection time (p = 0.7578), time of stimulus + collection time (p = 0.1204) and frequency + stimulus time + collection time (p = 0.9320).

For Experiments 1 and 2, discomfort scale was low and did not allow comparison between the currents and frequencies, as well, for induction and collection time of sweat. All controls showed normal levels of chloride and sodium in sweat test for Experiments 1 and 2. Skin temperature before stimulation was higher than after (p < 0.001 in both cases) (Tables 6 and 7). The layer of fat in the skin assessed by BMI showed no statistical difference for different frequencies and intensities of the currents, as well as different stimulation and collection times (p > 0.05). The CD ranged from 0.07 to 0.21 mA/cm^2^ (Table 8).

**Table 6:** **Frequency and current in relation to the skin temperature**

Frequency (Hz)*	Current ^#^	Skin temperature	Mean	Standard deviation
**0 (CDC)**	0.5	Before	30.9	0.4
		After	29.3	1.2
	0.75	Before	31.0	0.3
		After	30.6	1.5
	1	Before	30.5	0.9
		After	30.4	0.7
	1.5	Before	31.0	0.3
		After	30.6	1.5
**200**	0.5	Before	30.9	1.2
		After	29.6	0.4
	0.75	Before	31.5	3.2
		After	29.6	1.4
	1	Before	31.6	2.3
		After	31.2	1.8
	1.5	Before	29.4	1.8
		After	28.0	1.4
**1,000**	0.5	Before	31.8	0.9
		After	30.7	0.1
	0.75	Before	31.3	0.8
		After	30.1	0.8
	1	Before	30.4	2.3
		After	31.3	1.0
	1.5	Before	30.8	1.0
		After	31.1	2.0
**5,000**	0.5	Before	31.4	1.6
		After	29.3	1.4
	0.75	Before	30.2	0.8
		After	29.9	0.7
	1	Before	31.6	2.1
		After	30.3	2.1
	1.5	Before	32.8	1.5
		After	31.4	0.5

CDC, constant direct current; Hz, Hertz, mA, milliampere. *In the analysis considering the frequency in relation to skin temperature, p-value = 0.4102; ^#^In the analysis considering the current in relation to skin temperature, p-value = 0.5361; for interaction between current and frequency in relation to skin temperature, p-value = 0.0884. The skin temperature before the experiment was higher than after, p-value <0.0001.

**Table 7:** **Skin temperature considering frequency, time of stimulation and collection time, with fixed current of 1 mA**

Frequency (Hz)* ^,#^	Time of stimulation	Time of collection	Skin temperature	Mean	Standard deviation
**0 (CDC)**	10 min	30 min	Before	30.7	0.7
			After	30.2	0.8
		15 min	Before	31.1	2.3
			After	29.2	1.6
	5 min	30 min	Before	29.6	1.4
			After	29.3	1.4
		15 min	Before	31.6	1.7
			After	29.2	2.1
**200**	10 min	30 min	Before	31.3	1.1
			After	30.4	0.9
		15 min	Before	32.2	1.3
			After	31.3	2.3
	5 min	30 min	Before	32.3	1.1
			After	30.5	1.0
		15 min	Before	29.3	2.1
			After	28.2	3.5
**1,000**	10 min	30 min	Before	29.8	2.1
			After	30.1	1.6
		15 min	Before	31.6	2.0
			After	30.9	1.6
	5 min	30 min	Before	31.3	1.3
			After	29.6	0.5
		15 min	Before	31.9	2.1
			After	30.2	1.6
**5,000**	10 min	30 min	Before	31.2	2.8
			After	30.8	2.2
		15 min	Before	33.2	3.0
			After	30.9	2.8
	5 min	30 min	Before	29.9	0.9
			After	29.1	0.3
		15 min	Before	29.9	3.1
			After	29.2	2.2

CDC, constant direct current; Hz, Hertz; mA, milliampere. *In the analysis considering the frequency in relation to skin temperature with the time of stimulation, p-value = 0.4688; ^#^In the analysis considering the frequency in relation to skin temperature and collection time, p-value = 0.5770. For interaction between frequency + stimulation time + collection time, p-value = 0.4682. The skin temperature was significant higher before the sweat test than after, p-value <0.0001.

**Table 8:** **Current densities (electrode with 3 cm of diameter)**

Current (mA)	Current density (mA/cm ^2^)	% volume of sweat above 54 mg*
0.5	0.07	12/12 (100%)
0.75	0.12	12/12 (100%)
1.0	0.14	12/12 (100%)
1.5	0.21	12/12 (100%)

*The adequate sweat rate was considered for an area practically of 18 cm^2^ and 30 min of sweat stimulation. For the classical Gibson & Cooke test, the area considered was related of a 5.5 cm diameter filter paper with 30 min of stimulation, and the 75 mg criteria was used as the gold standard for the sweat.

### Experiment 3 – classical and atypical CF

**Figure 3:**
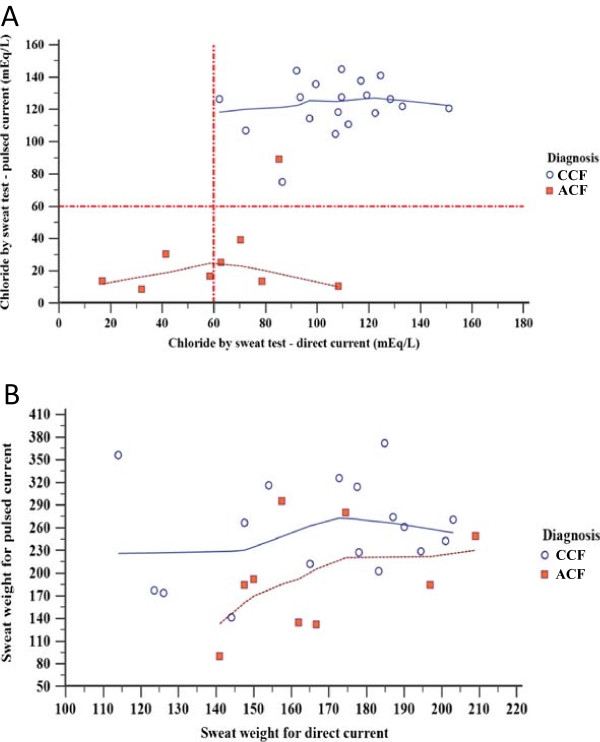
**Pulsed direct current and constant direct current in typical and atypical cystic fibrosis (CF) patients considering the CF diagnosis and sweat weight. (A)** Linear regression for chloride values (mEq/L) taking into account constant direct and pulsed direct currents. In the graphic is shown the cystic fibrosis diagnosis by *CFTR* screening and rectal biopsy (in blue color). Twenty-eight subjects were enrolled in the study. The correlation coefficient was 0.704, 95% CI: 0.449 to 0.853 (p < 0.001). For the same graphic there was no association between the two sweat tests performed [pulsed direct (92.05 ± 49.28) and constant direct currents (92.80 ± 31.84)] for the values of the Chloride (mEq/L) (p = 0.911). Pared T-test and Spearman’s correlation test (α = 0.05) were used to study the association and correlation between variables, respectively. **(B)** Linear regression for sweat weight values (mg) taking into account constant direct and pulsed direct currents. The cystic fibrosis diagnosis by *CFTR* screening and rectal biopsy (in blue color) were shown in the graphic. Twenty-eight subjects were enrolled in the study. The correlation coefficient was 0.213, 95% CI: −0.190 to 0.554 (p = 0.296). For the same graphic, we observed higher values for the sweat weight on pulsed direct current (234.86 ± 72.49) than the constant direct current (167.34 ± 25.79) (p = 0.001). For the association was used the test T pared and for the correlation the Spearman test. α = 0.05. CCF, Classical Cystic Fibrosis; ACF, Atypical Cystic Fibrosis.

Both the CDC (p = 0.006) and PDC (p < 0.001) showed values consistent with the results of rectal biopsy and *CFTR* mutations. However, the Kappa index was higher for PDC (Table 9). PDC showed correlation on chloride levels with CDC (Figure 3A); the same was not true for the sweat weight (Figure 3B).

**Table 9:** **Sweat test diagnosis by constant direct and pulsed direct currents taking into account cystic fibrosis diagnosis by other methods**

Sweat test	Groups	CF diagnosis*		Total	p-value	Cohen’s Kappa index	Standard error	Agreement
		Typical CF	Atypical CF					
Constant direct current	Typical CF	19	5	24	0.006	0.5205	0.1658	Moderate
	Atypical CF	0	4	4				
Total		19	9	28				
Pulsed direct current	Typical CF	19	1	20	<0.001	0.9218	0.1851	Almost perfect
	Atypical CF	0	9	9				
Total		19	10	29				

*CF diagnosis was performed by *CFTR* mutation screening and CFTR activity in rectal biopsies by Ussing chamber. The first sweat test performed in the subjects was done by constant direct current; golden standard diagnosis tool. The pulsed direct current was done to compare the results with the constant direct current and the *CFTR* mutations and CFTR expression and function by rectal biopsies. All subjects enrolled have clinical symptoms related with CF disease.

## Discussion

Although CST is considered the golden standard for the CF diagnosis [3, 4, 6, 23–25], studies have reported difficulties in performing the test, especially for sweat collection and electrolyte measurement [26–28]. The sweat stimulation, which is the first step for CST, had few studies since its implementation and standardization, in 1959, by Gibson and Cooke [2], and was enhanced in 1983 by the Webster Sweat inducer system [29], along with the Wescor Macroduct kit [14, 30]. Although the benefits of iontophoresis have been exploited for other purposes, the CST has not been changed.

Advantages of the PDC use for the iontophoresis method over CDC has been demonstrated in studies with transdermic delivery of substances [18, 31–34]. Most studies showed greater efficacy of the PDC for delivery of substances into the skin [2, 29].

PDC iontophoresisis is less uncomfortable than the CDC. The depolarization occurs between pulses, preventing epidermal irritation [33, 35, 36]. In the present study, in healthy adults, both PDC and CDC were not annoying. This finding may be due to the use of low CD used for the PDC and CDC.

The CDC can cause permanent polarization of the skin during the stimulation, which affects the efficiency of substance delivery proportionally to the time of application of the impedance. This fact can be minimized by the use of PDC, which is delivered periodically [37, 38]. During the phase without stimulation, the skin becomes depolarized and returns to its initial polarization. However, Bagniefski and Burnett (1990) showed that greater depolarization of the skin may decrease the efficiency of drug delivery, if the frequency is high [18]. In this study, frequencies of 1,000 to 5,000 Hz were more effective for pilocarpine delivery and induction of skin perspiration for the CST compared to lower frequencies of zero to 200 Hz. In our study, all subjects in the Experiment 1 had sufficient sweat weight (< 54 mg) for low and high frequencies.

Studies with PDC have shown that the waveform influence on the permeation of the substance into the skin. Chien and Chen [39] had studied the absorption of luteinizing- hormone-releasing hormone (LHRH) using CDC and PDC with different waveforms. Sinusoidal and rectangular shapes waves were equal to CDC in the flux of hormone delivery, but the flow for triangular waves was lower than the CDC. Chien et al. [40] showed that the flow of drug delivery was efficient for sinusoidal, trapezoidal and rectangular waves. Hirvonen [41] reported that the amino acids permeability into skin by CD of 0.5 mA/cm^2^ and frequency of 2.5 kHz were equal of rectangular and sinusial waves. Kanebako [42] showed that the permeability of the drug tested was better with the use of PDC with frequencies below 100 Hz and rectangular wave.

The square waveform was tested by Panzade et al. [38] for granisetron penetration by iontophoresis, and showed better results in CDC. However, the PDC was considered less uncomfortable to the skin.

Unlike previous studies, we use the PDC with triangular waveform and lower current values, which resulted in adequate sweat weight. In this case, the PDC was a useful procedure to stimulate sweating. This finding should bring significant benefits, especially for younger children.

The substance penetration into the skin depends on the time for the applied current in iontophoresis [43]. Recent consensus on CST suggested that the stimulation time of 5 min is capable to stimulate sweating [6, 7, 13, 44]. In our experiments, 10 min of stimulation was able to induce sweat levels greater than 54 mg, which was better than 5 min. This fact is consistent with the first study inducing sweat iontophoresis [2]. In the present study, Experiment 2 showed 41.7% (10/24) of subjects with insufficient sweat weight (< 54 mg) for stimulation time of 5 min and 4.2% (1/24) for 10 min (p = 0.002). In this case, the pilocarpine amount increased with the time of stimulation.

It is well known that sweat is not to be collected longer than 30 min and not less than 20 min [7, 45]. In the present study, the greatest weight in sweat occurred with 30 min of collection, with CD of 0.14 and 0.21 mA/cm^2^.

Another important aspect related with the sweat test is the CD. Wrong CD calculation can cause risk of skin irritation and burns, therefore the maximum CD tolerability for the human skin is of 0.5 mA/cm^2^ [37, 38, 46, 47]. If the CD increases, the flow also increases, and consecutively the substance delivery. In the study of Panzade et al. [38], CDC proved to be 3.8 times higher for substance delivery compared to PDC. Then, the increase in CD causes an increase in transport of the drug through the pores of the skin.

In studies that induce sweat with pilocarpine it is not possible to identify the values of CD. Two guideline suggested 0.16 to 0.24 mA/cm^2^ as suitable CDs to CST [44, 48]. In our study, the CDs between 0.14 to 0.21 mA/cm^2^ were efficient for pilocarpine delivery into the skin by iontophoresis, inducing stimulation of the sweat in 100% of subjects with stimulation time of 10 min and 30 min for collection.

One of the disadvantages of substance delivery into skin by iontophoresis is the risk of shocks and burns due to the exposure of an electric current in the skin for long periods, overdose, lesions at the site of application, alternation of the pH and high current intensities [49, 50].

In our study, no subject had significant side effects, especially due to the low DC used, and indicating great safety.

The skin has relatively high impedance, which is mainly associated with the stratum corneum [45, 51]. During the iontophoresis, skin resistance decreases and ion concentration increases in stratum corneum, allowing the substance delivery during electric current stimulation. At the end of the current flow, the ion concentration in the skin returns to physiological levels [52]. There is no evidence that the current amplitude and stimulation time are associated with temporary decrease in impedance, or with reduction in skin protection [53]. In the present study, with the stimulation times of 5 and 10 min, no differences in the impedance of the electrode, gauze and skin system were observed.

Bioelectric properties of the skin allow the application of various forms of electric current. The opposition offered by the biological tissue is called impedance present in inter- and extracellular fluid, and the capacitive reactance of cell membranes. To occur the absorption of substances through the skin, the electrical current must overcome the impedance imposed on their flow and reach the target tissue with sufficient intensity [54, 55]. Adipose tissue is bad conductor of electrical current, causing an increase in impedance. Although some studies have reported that the frequency variation would not influence the decrease in skin impedance [18, 42, 56], Chien et al. [40] reported that the skin impedance declined with increasing current frequencies. In our study, PDC with a frequency of 1,000 Hz showed lower impedance than CDC, providing adequate sweat production. Additionally, the subjects enrolled did not show high BMI, which could reduce the pilocarpine delivery and reduction of sweat production.

Electric current follows the path of minor resistance, so skin impedance determines its intensity, density and path, altering the biological responses. Skin impedance changes with the distance between electrodes, positioning, location of the electric field, amount of water and number of layers of the stratum corneum. Keratin is the main barrier to the passage of electric current [57–63].

The interelectrode distance adopted in this study was of 2 inches and was suitable for sweat stimulation. As the ambient temperature can lead to decreased hydration of keratin and thickness of biological tissue, and thereby influence the impedance increase in skin [64], the room temperature was maintained at 25°C.

Temperature increase at the electrode is proportional to the square of CD [61]. This effect was measured on the place of the skin contact with the electrodes, before and after stimulation. The applied CD promoted cooling, and no heating of the electrodes, preventing burns.

Our study showed that, despite the wide CST use for the CF diagnosis, there is still a field of studies to assess and maximize the efficiency and effectiveness of stimulation and collection steps. Currently, there is an increasing frequency of the CST use, especially in younger individuals, due to the implementation of CF newborn screening in our country. Therefore, we believe that the use of PDC may be a benefit for infants in the first year of life due to lower risk and discomfort associated.

## Conclusion

CST with CDC or PDC using CD of 0.07 to 0.21 mA/cm^2^ showed efficacy in stimulation and collection steps of sweat test without side effects and adequate sweat weight to perform the CST, with a greater concordance of CF diagnosis for PDC sweat test.

## Electronic supplementary material

Additional file 1: **Visual analogue scale.** (JPEG 10 KB)

Additional file 2: **Genotypes for the** ***CFTR*** **mutations of typical and atypical cystic fibrosis patients enrolled in the study (n = 29).** (DOCX 13 KB)

Additional file 3: ***CFTR*** **mutations found in individuals enrolled in the study.** Gene and protein localization. Mutation classification and frequency from the present study are designated. Traditional and HGVs standard nomenclature for *CFTR* mutations are also indicated. (DOCX 15 KB)

## Abbreviations

CDC: Constant direct current
PDC: Pulsed direct current
Cl^−^: Chloride
CD: Current density
VAS: Visual analogue scale
CF: Cystic fibrosis
I: Current
BMI: Body mass index
I_rms_: Effective measured current
J: Density
LHRH: Luteinizing-hormone-releasing hormone
MIM: Mendelian inheritance in man
S: Electrode area
CST: Classic sweat test
V_rms_: Voltage
Z: Composite impedance.
