# Supplementary figures and images for: Pulsed direct and constant direct currents in the pilocarpine iontophoresis sweat chloride test
Source: BMC Pulm Med. 2014 Dec 13;14:198. doi: 10.1186/1471-2466-14-198 (PMC4290820; doi:10.1186/1471-2466-14-198)

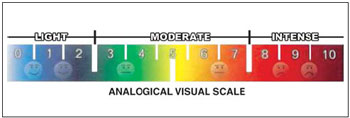

Supplement: Supplementary file 1 — Additional file 1: Visual analogue scale. (JPEG 10 KB) [file 12890_2014_641_MOESM1_ESM.jpeg]
