# Supplementary material for: Pulsed direct and constant direct currents in the pilocarpine iontophoresis sweat chloride test
Source: BMC Pulm Med. 2014 Dec 13;14:198. doi: 10.1186/1471-2466-14-198 (PMC4290820; doi:10.1186/1471-2466-14-198)
Supplement: Supplementary file 2 — Additional file 2: Genotypes for the CFTR mutations of typical and atypical cystic fibrosis patients enrolled in the study (n = 29). (DOCX 13 KB) [file 12890_2014_641_MOESM2_ESM.docx]

| **Supplementary 1.** Genotypes for the *CFTR* mutations of cystic fibrosis patients enrolled in the present study (n= 29) | | | |
| --- | --- | --- | --- |
| **Mutation** | **Numberofpatients** | **Percentage (%)** | **Cumulative percentage** |
| F508del/F508del | 3 | 10.34 | 10.34 |
| F508del/G542X | 2 | 6.90 | 17.24 |
| F508del/c.1717-1G>A | 1 | 3.44 | 20.68 |
| F508del/3272-26A>G | 1 | 3.44 | 24.12 |
| 3120+1G>A/R1066C | 1 | 3.44 | 27.56 |
| 3120+1G>A/L206W | 1 | 3.44 | 31 |
| F508del/P205S | 1 | 3.44 | 34.44 |
| F508del/R1066C | 1 | 3.44 | 37.88 |
| F508del/S549R | 1 | 3.44 | 41.32 |
| A561E/A561E | 1 | 3.44 | 44.76 |
| G542X/2183AA>G | 1 | 3.44 | 48.2 |
| G542X/I618T | 1 | 3.44 | 51.64 |
| G542X/R334W | 1 | 3.44 | 55.08 |
| G576A/NMI | 1 | 3.44 | 58.52 |
| F508del/NMI | 2 | 6.90 | 65.42 |
| NMI/NMI | 10 | 34.48 | 100 |

*CFTR* = Cystic Fibrosis Transmembrane Regulator; N = number of patients, NMI = No mutation identified.
