# Supplementary material for: Pulsed direct and constant direct currents in the pilocarpine iontophoresis sweat chloride test
Source: BMC Pulm Med. 2014 Dec 13;14:198. doi: 10.1186/1471-2466-14-198 (PMC4290820; doi:10.1186/1471-2466-14-198)
Supplement: Supplementary file 3 — Additional file 3: CFTR mutations found in individuals enrolled in the study. Gene and protein localization. Mutation classification and frequency from the present study are designated. Traditional and HGVs standard nomenclature for CFTR mutations are also indicated. (DOCX 15 KB) [file 12890_2014_641_MOESM3_ESM.docx]

| **Table 2** *CFTR* mutations found in individuals under study. Gene and protein localization. mutation classification and frequency from the present study are designated. Traditional and HGVS standard nomenclature^a^ for *CFTR* mutations are also indicated | | | | | | | |
| --- | --- | --- | --- | --- | --- | --- | --- |
| Traditional Nomenclature | HGVS Nomenclature^a^ | | Localization (*CFTR* gene)^d^ | Consequence | Protein localization | Mutation Classification | Predicted Functional Class |
|  | cDNA name | Protein name |  |  |  |  |  |
| F508del | c.1521_1523delCTT | p.Phe508del | Exon 10 | Point deletion | NBD1 | A | II |
| G542X | c.1624G>T | p.Gly542X | Exon 11 | Nonsense | NBD1 | A | I |
| R334W | c.1000C>T | p.Arg334Trp | Exon 7 | Missense | TM6 | A | IV |
| 3120+1G>A | c.2988+1G>A | - | IVS16 | Splicing | - | A | I |
| P205S | c.613C>T | p.Pro205Ser | Exon 6a | Missense | TM3 | A | IV |
| 1717-1G>A | c.1585-1G>A | - | IVS11 | Splicing | - | A | I |
| 2183AA>G | c.2051_2052delAAinsG | p.Lys684SerfsX38 | Exon 13 | Frameshift | RD | A | I |
| A561E | c.1682C>A | p.Ala561Glu | Exon 12 | Missense | NBD1 | A | II |
| 3272-26A>G | c.3140-26A>G | - | IVS17b | Splicing | - | A | V |
| I618T | c.1853T>C | p.Ile618Thr | Exon 13 | Missense | NBD1/ RD | A | IV |
| R1066C | c.3196C>T | p.Arg1066Cys | Exon 17b | Missense | ICL4 | A | II |
| S549R(T>G) | c.1647T>G | p.Ser549Arg | Exon 11 | Missense | NBD1 | A | III |
| L206W | c. 617T>G | p.Leu206Trp | Exon 6a | Missense | TM3 | A / B | IV |
| G576A | c.1727G>C | p.Gly576Ala | Exon 13 | - | NBD1/ RD | B | - |

A - CF-causing mutation; B - CFTR-RD mutation.

^a^ Reference CFTR sequence accession number: NM_000492.3, nucleotide number 1 corresponds to the A of the ATG translation initiation codon, in the reference sequence is numbered as 133.

^b^ According to the HVGS guidelines this mutation should be named: 1585-9412bp A>G.

^c^ Inclusion of 104bp criptic exon between exon 10 and exon 11 in the CFTR transcripts.

^d^ Traditional Nomenclature.
